# Seagrass Leaves: An Alternative Resource for the Production of Insulation Materials

**DOI:** 10.3390/ma15196933

**Published:** 2022-10-06

**Authors:** Aldi Kuqo, Carsten Mai

**Affiliations:** Department of Wood Biology and Wood Products, Faculty of Forest Sciences and Forest Ecology, University of Goettingen, Büsgenweg 4, 37077 Göttingen, Germany

**Keywords:** fire properties, *Posidonia oceanica*, seagrass wracks, thermal insulation, waste valorization, *Zostera marina*

## Abstract

Seagrass wracks, the remains of dead leaves accumulated on seashores, are important ecosystems and beneficial for the marine environment. Their presence on the touristic beaches, however, is a problem for the tourism industry due to the lack of aesthetics and safety reasons. At the present time, seagrass leaves are landfilled, although this is not considered an ecological waste management practice. Among other proposed practices for more sustainable and environmentally friendly management, such as composting and biogas or energy generation, in this study we aim to use seagrass leaves for the production of insulation materials. Insulation boards from two types of seagrass leaves (*Posidonia oceanica* and *Zostera marina*) at densities varying from 80 to 200 kg m^−3^ were prepared and their physical and mechanical properties were examined and compared to those of wood fiber insulation boards. The thermal conductivity of seagrass-based insulation boards varied from 0.042 to 0.050 W m^−1^ K^−1^, which was up to 12% lower compared to the latter. The cone calorimetry analysis revealed that seagrass-based insulation boards are more fire resistant than those from wood fibers, as they release very low amounts of heat during combustion and do not ignite when exposed to a single flame (Bunsen burner). A simplified cost analysis showed that insulation boards made from seagrass leaves can be up to 30% cheaper compared to those made from wood fibers. After their end of life, seagrass leaves can again be considered a valuable resource and be further utilized by adopting other management strategies.

## 1. Introduction

Seagrasses are marine flowering plants that can form underwater meadows. Their presence can extend up to 90 m below sea level [1]. The global distribution of seagrass is estimated at 177,000–600,000 km^2^ [2]. Seagrass species come in many different shapes and sizes. Their leaf size can vary from several cm up to 7 m. The largest amount of seagrass is found on the shores of Australia, but also on the continental coasts of the Americas, northern Europe, and more abundantly in the Mediterranean area [2]. According to Cebrian and Duarte [3], a moderately wide belt of seagrass may yield more than 125 kg of dry material per square meter of the coastline each year. Although seagrasses are crucial habitats for many marine organisms [4], their leaves break off after their growing season and are moved by wave currents, settle on the shores, and start decomposing. The seagrass leaves undergo microbial breakdown, emitting greenhouse gases such as carbon dioxide (CO_2_) and methane (CH_4_) into the atmosphere [5,6]. Apart from the release of greenhouse gases and unpleasant smells due to natural degradation, the effects of seagrass wrack lying on beaches include the impairment of tourism in the affected areas. Seagrass wrack is therefore removed from the shoreline and disposed of in landfills [7,8,9,10].

Landfilling is considered the least effective way to manage this waste biomass according to the European Union (EU) Waste Framework Directive [11]. For sustainable management, a paradigm shift should be made by no longer considering seagrass wrack as a waste, but as a resource. Thus, much research has been carried out to study the various ways of valorizing seagrass wrack. A current study conducted by Mainardis et al. [12] indicated that composting is one of the most effective ways to utilize organic seagrass residues. In another work, the material has been studied to unveil its potential for biogas production in the anaerobic digestion process [8,13]. Other researchers have taken different approaches to solving the problem of seagrass waste utilization. Khiari and Belgacem [14] studied the possibility of producing cellulose pulps and paper. It has already been confirmed that this raw material has potential for paper production. The high content of mineral components, however, might have a negative effect on the chemical recovery process in the papermaking industry [14]. Another potential use of seagrass reported in the literature is its conversion into energy [15]. Converting the waste into a carbon-neutral biocoal is also a viable alternative [16].

The conversion of seagrass waste (leaves) into a functional material for the construction sector is one of the most frequent approaches for its utilization. Much work has focused on the use of seagrass leaves bonded with organic binders (pMDI, UF, etc.) to produce medium-density fiber/particleboards [17,18]. In other cases, researchers have used mineral binders such as cement for the production of insulation composites [19]. Although the relatively high-density organically or minerally bonded seagrass-based materials seem to be an interesting alternative to conventional wood fiberboards/particleboards; their low mechanical properties are a major drawback that limits their application. The aim of our study is to investigate the use of seagrass leaves extracted from seagrass wracks for the production of low-density, organically bonded insulation materials, and to compare them with those made from wood fibers. It should be noted that one of the seagrass types investigated in this study, *Posidonia oceanica*, which occurs in the Mediterranean area, is subdivided into seagrass fibers and leaves. Seagrass fibers have been extensively studied and are already commercialized as an insulation material [10,20,21]. Leaves, however, are less investigated in this regard. Along with *Posidonia oceanica* leaves (POL), the second species of seagrass studied is *Zostera marina* (ZM). ZM seagrass is widely distributed in the Northern Hemisphere [22]. ZM leaves were applied in their loose form as insulation material (Cabot’s Quilt) at the beginning of the 20th century [23]. Rather than a loose-fill or a blow-in insulation material, our objective is to produce and characterize rigid boards intended for partition walls, ceilings, and roofing. Our motive for conducting this research was not only to investigate a feasible approach to seagrass waste management, but also to produce a low-cost building product that can be applied for insulation.

A well-insulated building is a precondition for an economically viable use of energy. Driven by governmental measures to reduce greenhouse gas emissions, improve cost efficiency, and adopt new regulations for energy-efficient buildings, global demand for thermal insulation materials in building applications is projected to increase. Wood fiber insulation boards have been trending lately and are regarded as laudable alternatives to synthetic insulation materials [24,25]. However, the high energy demand for refining wood fibers and the scarcity of raw wood in particular areas have initiated research into substitute resources.

Apart from the initial morphological and chemical characterization of raw materials and the examination of the physico-mechanical properties of the boards produced, a simplified cost analysis was carried out to estimate the economic profitability of using seagrass leaves compared to wood fibers for the production of insulation boards.

## 2. Materials and Methods

### 2.1. Materials

*Zostera marina* seagrass leaves were provided by Seegrashandel GmbH (Westerau, Germany). The raw material had been collected in the East Sea. After natural drying under ambient conditions, leaves with a length of 5 to 60 cm were cut to shorter lengths to avoid problems during the spraying process. Leaves of the Mediterranean seagrass *Posidonia oceanica* were collected on the shore of Durrës, Albania. The relatively freshly washed-up seagrass was collected in December 2021. The leaves were dried and shaken several times to remove excess sand before further processing. Wood fibers (a mixture of spruce and pine wood fibers) for the production of reference boards were provided by GUTEX GmbH (Waldshut-Tiengen, Germany). A low-temperature curing polymeric methylene diphenyl diisocyanate (pMDI) resin (I-Bond WFI 4370, Huntsman, Everberg, Belgium) was used as a binder.

### 2.2. Methods

#### 2.2.1. Determination of Morphological Characteristics

The geodesic length and thickness density distribution weighted by surface area (q2) of seagrass leaves and wood fibers were measured using FibreShape PRO (X-shape, IST, Vilters, Switzerland). Representative samples of *Posidonia oceanica* leaves (3.5 g), *Zostera marina* leaves (2.0 g), and wood fibers (0.1 g) were manually dispersed on a transparent film of A4 size. The raw leaves and fibers were scattered in such a way that they did not overlap. High-resolution images were created using a flatbed scanner (Epson Perfection V850 Pro, Epson, Tokyo, Japan) in transmitted light mode. The object dimensions were assessed by the static image analysis of the FiberShape software. The internal structure of seagrass leaves was scanned using a digital 3D-reflected light microscope VHX-5000 (Keyence, Neu-Isenburg, Germany).

#### 2.2.2. Chemical Analysis

The laboratory analytical procedures (LAP) of the National Renewable Energy Laboratory (NREL) and the standards published by the Technical Association of the Pulp and Paper Industry (TAPPI) were used to determine the chemical composition of the raw lignocellulosic materials. Initially, the material (seagrass leaves and wood fibers) was ground in a cutting mill (Retsch GmbH, Haan, Germany) with a 0.5 mm screen. Each analysis step was performed in duplicates. The first step of the cascading procedure was the hot-water extraction. The oven-dried lignocellulosic material was extracted for 5 h in a Soxhlet extractor and then dried at 103 °C. Afterward, an ethanol–cyclohexane (1:2) extraction was conducted for another 5 h. The tests were performed according to T264 cm-97 [26]. NREL/TP-510-42618 LAP [27] was used to determine the lignin content of the extractive free material. After the hydrolysis of the polysaccharides with sulfuric acid (72%), the remaining solids (lignin) were weighed after rinsing with demineralized water and drying. The holocellulose content was determined according to the procedure established by L. E. Wise [28]. The evaluation of the ash content was conducted according to the procedure described in TAPPI T 211 [29].

#### 2.2.3. Production of Insulation Boards

**Figure 1:**
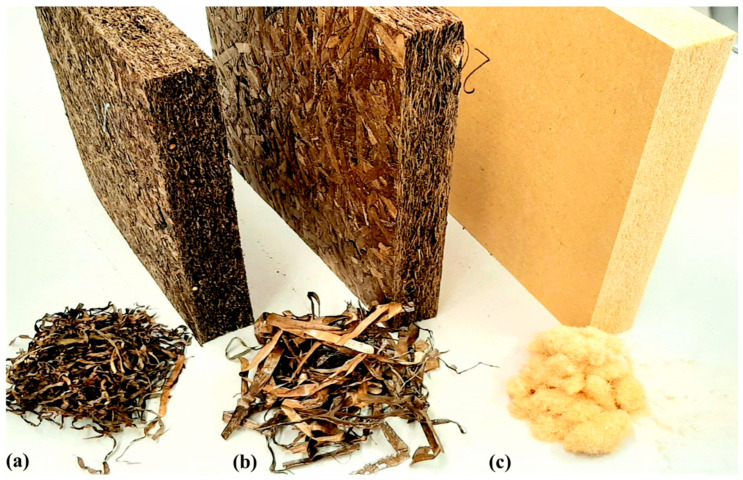
Seagrass boards and the respective raw materials. Boards produced from *Zostera marina* leaves (**a**), *Posidonia oceanica* leaves (**b**), and wood fibers (**c**).

The boards were produced by using a dry process. The raw material was vigorously stirred in a gluing drum and the adhesive sprayed through a nozzle at a flow rate of approximately 0.1 to 0.15 g s^−1^. Before further processing, the moisture content was determined thermogravimetrically. The moisture content of naturally dried *Zostera marina* leaves was 13.9%; for *Posidonia oceanica* leaves it was 15.5%, while for wood fibers it was as high as 9.2%. The adhesive proportion for the boards produced was 6 wt%. The sprayed material was cold pre-pressed and then hot-pressed at 190 °C. Before hot-pressing, 30 g of water was sprayed onto each side of pre-pressed boards to allow steam to form and activate the binder. The dimensions of the boards were 450 × 450 × 40 mm^3^. In total, 24 boards (8 boards for each type of raw material for 4 different target densities) were prepared (Figure 1, Table 1). The boards were cut to test specimen dimensions and conditioned to constant mass at 20 ± 2 °C and 50 ± 5% RH prior to testing.

#### 2.2.4. Mechanical Testing

Specimens whose density was within ±10% of the target density were selected for the mechanical tests. For the compression test, a compressive force was applied perpendicular to the faces of the test specimen at a constant speed (0.1 d min^−1^ ± 25%, in which d is the thickness of the test specimen in mm) using a universal testing machine (ZwickRoell Zmartpro, ZwickRoell, Ulm, Germany) with a 10 kN load cell. The tests were conducted according to DIN EN 826 [30]. Four specimens per variant with nominal dimensions of 50 × 50 × 40 mm^3^ were tested. The internal bond strength (tensile strength perpendicular to the faces) was evaluated following DIN EN 1607 [31]. The test specimens (with nominal dimensions of 50 × 50 × 40 mm^3^) were glued between two stiff boards with a fast-curing polymer adhesive and then inserted into the universal testing machine. Tensile stress was applied at a constant speed of 10 mm min^−1^. Four specimens were tested for each variant.

#### 2.2.5. Thermal Conductivity Measurements

Thermal conductivity was determined following the procedure described in the standard DIN EN 12667 [32]. The Heat Flow Meter HFM 446 Lambda Eco-Line (NETZSCH Group, Selb, Germany) was used for the measurement. Two specimens for each variant (target density) measuring 250 × 250 × 40 mm^3^ were tested at 5 different temperatures (10, 15, 20, 25, and 30 °C). The equipment quantifies the steady-state heat flow through a test specimen placed between two plates with thermal sensors. The difference between the hot and cold plates was 20 °C. The thermal conductivity for 23 °C (λ_23_) was calculated by applying linear regression.

#### 2.2.6. Cone Calorimetry and Single Flame Tests

The procedure described in ISO 5660-1 [33] was adopted for the cone calorimetry test. Tetragonal specimens (100 × 100 mm^2^) with a given thickness were exposed to a heat flux of 50 kW m^−^² for 30 min. The peak heat release (PHR), the total heat release after 1800 s (THR), and the mass loss rate at the first 300 s (MRL) were evaluated using a mass loss calorimeter (MLC FTT, Fire Testing Technology, East Grinstead, UK). In addition, the ignition and the flameout times were assessed. Three specimens were tested for each variant.

The single flame test was conducted according to DIN EN ISO 11925-2 [34]. Specimens sized 250 × 90 × 40 mm^3^ were clamped to the fire chamber device (Taurus Instruments AG, Weimar, Germany). Their surface was exposed to fire for 15 s. The flame of the Bunsen burner had a flame height of 20 mm. The direction of flame was 45° to the direction of the specimen. After flame exposure, the pyrolytic behavior of the sample was evaluated by observing whether ignition took place and if the flame reached a height of 150 mm above the burning point in the specified time span; the soot cone height (height of burned area) was measured. An additional set of specimens was tested following the same procedure, with the lower edges of the specimens exposed to the flame for 30 s. Two specimens were tested for each variant.

#### 2.2.7. Water Absorption Test

The water absorption test was determined following the standard EN 1609 [35] with minor modifications. Specimens measuring 100 × 100 × 40 mm^3^ were placed in the empty water vessel and remained partially submerged as water was added. The water was carefully filled into the container until the underside of the specimen was (10 ± 2) mm below the water level. After 24 h immersion in water, the specimens were weighed and the increase in weight per square meter after submersion was calculated. In total, 3 specimens were tested for each variant.

## 3. Results and Discussion

### 3.1. Morphological Considerations and Chemical Composition

Seagrass has long, flattened leaves that are thin and blade-like. Wood fibers, in contrast, have a fibrous, structure with irregular (ideally round) cross-sectional shapes. Assuming that wood fibers have a cylinder-like structure, their shape can be easily examined as the thickness of the analyzed object corresponds to their diameter. During the test, seagrass leaves were preferentially positioned in the scan bed. The length and width of seagrass leaves can be easily determined. Their thickness (third dimension), however, cannot be measured from the 2D scan. The thickness of seagrass leaves was measured by examining the microscopy images. The POL leaves exhibited a thickness that varied from 100 to 200 µm, while ZM leaves appeared to be slightly thinner, having a thickness of 80 to 150 µm. The static image analysis showed the major differences between seagrasses and WF in terms of geodesic length and width (thickness for fibers) (Table 2).

The geodesic length of POL leaves varied from 5.2 to 99.7 mm. ZM leaves were slightly shorter compared to the former, reaching up to 86.01 mm. WF were seemingly short, up to 16.61 mm long, while their median geodesic length was 2.1 mm. It must be noted that wood fibers might contain a large proportion of fines, which differs greatly from the median geodesic length of seagrass leaves (Table 2). As indicated by FiberShape, POL leaves were wider compared to ZM. It is worth noting that some seagrass leaves may fold, resulting in reduced dimensions.

In terms of their chemical composition, seagrass leaves contain a higher content of extractives compared to wood fibers (Table 3). A high content of extractives in POL leaves has also been reported in previous studies [14]. In the case of ZM leaves, it has previously been reported that their content of polysaccharides is high while their lignin content is low. ZM leaves’ composition is comparable to sisal and jute [36]. A significant difference in lignin content was found between seagrasses and wood fibers. The latter contained 28.3% lignin, which is significantly higher compared to the former. According to Khiari and Belgacem, the differences in terms of lignin and holocellulose content are likely related to climate conditions and the chemical composition of the soil [14]. The ash content of seagrasses was considerably high and similar to that of rice and flax shives [37,38]. The high ash content is attributed to the chemical composition of the marine environment in which the plants grow and/or to their contamination by sand. Further elemental analysis showed that the main element of ash is silicon [14].

### 3.2. Mechanical Properties of Insulation Boards

Owing to the different chemical composition and morphological features of the lignocellulosic materials, different internal bond and compression strengths of the respective boards were obtained (Table 4). The internal bond strength tended to increase with increasing target density for all variants. ZM and POL boards displayed three to four times lower internal bond strength compared to WF boards.

During the pressing process, the long seagrass leaves tend to position themselves preferentially in a horizontal direction. The flat and wide surfaces of leaves are attached, forming a compact, multilayer structure. When transversal stress is applied, the bonded leaves can break, resulting in mechanical failure. At high densities, as a result of high compaction, increasingly more leaves are attached and glued to each other, increasing the bonded surface. In the case of WF boards, along with the chemical bonding provided by the pMDI binder, the wood fibers are interlocked with each other to form a stable structure. In addition, shorter and thinner fibers usually provide high internal bond strength [39].

The compression strengths at 10% deformation showed a similar trend with the internal bond strength as the target density increased. WF boards exhibited higher compression strength compared to the corresponding POL and ZM boards. In the case of the former, wood fibers are interlocked with each other and positioned in various directions on the board. The bundles of the randomly directed wood fibers “resist” the applied vertical force, leading to high compression strengths. During the production of seagrass boards, seagrass leaves lay horizontally. A portion of the leaves, however, tended to fold, resulting in the formation of large voids within the POL and ZM board structure. These large voids are responsible for the low compression strength. The higher compression strength of WF boards can be explained by the higher glue effectiveness as well. Short and thin wood fibers have a great surface area and glue is utilized to a high extent [39].

### 3.3. Thermal Conductivity of Insulation Boards

**Figure 2:**
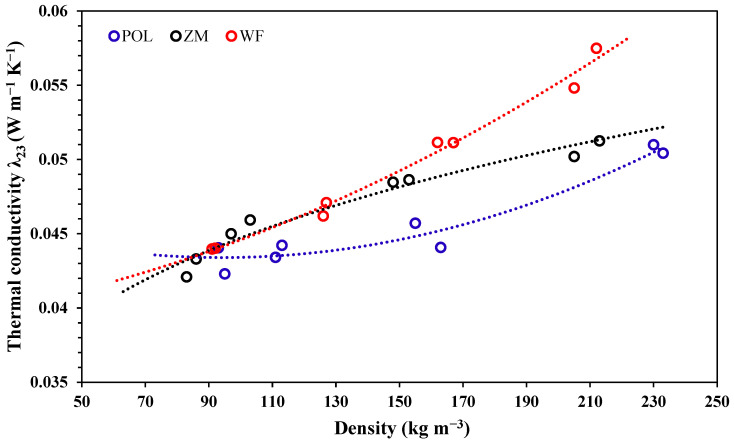
Thermal conductivity of seagrass-based boards (POL and ZM) and wood fiber-based boards (WF) at 23 °C for actual densities varying from 80 to 228 kg m^−3^.

Thermal conductivity λ (TC) is a measure of the effectiveness of a material in conducting heat. The investigation of this property allows a quantitative comparison between the effectiveness of different thermal insulation materials. The TC of produced boards depended on their actual density (Figure 2). POL boards exhibited the lowest TCs, varying from 0.042 to 0.050 W m^−1^ K^−1^. ZM boards had slightly higher values of TC compared to POL boards but were still within the same range. WF boards seem to conduct thermal energy better, as TC was higher in their case and varied from 0.044 to 0.057 W m^−1^ K^−1^. The fitted curves associated with TC values illustrate the differences between the produced boards (Figure 2). It is evident that POL boards have a substantially lower TC at relatively high densities, ranging from 150 to 228 kg m^−3^, compared to other variants. For WF boards, there is a strong increase in TC with density (stronger than that of the other variants).

The amount and volume of voids between the lignocellulosic aggregates in the boards decrease considerably with increasing board density. The heat flow is transferred through the solid material (conduction) and air voids (convection). The TC of air within the voids is lower than that of solid material; thus, the resulting material has low TC at decreasing density. Apart from the chemical composition, another cause for the low TC of seagrass-based boards might be the shape and size of seagrass leaves. During the pressing operation, the seagrass leaves lie longitudinally. Both seagrass-based boards are composed of multiple individual layers that are bonded with each other. Heat is conducted through the solid material mainly in the longitudinal direction, but it can be also transferred from one layer to another through conduction (if the layers are attached to one another). Many voids are also present between the layers of seagrass leaves. In this case, the heat is transferred through convection. Overall, the heat transfer in the vertical direction is low. WF boards, on the other hand, contain fiber bundles that are extended and a large proportion of which are also vertically directed. The heat is effectively conducted through the wood fibers, which eventually leads to high TC.

**Figure 3:**
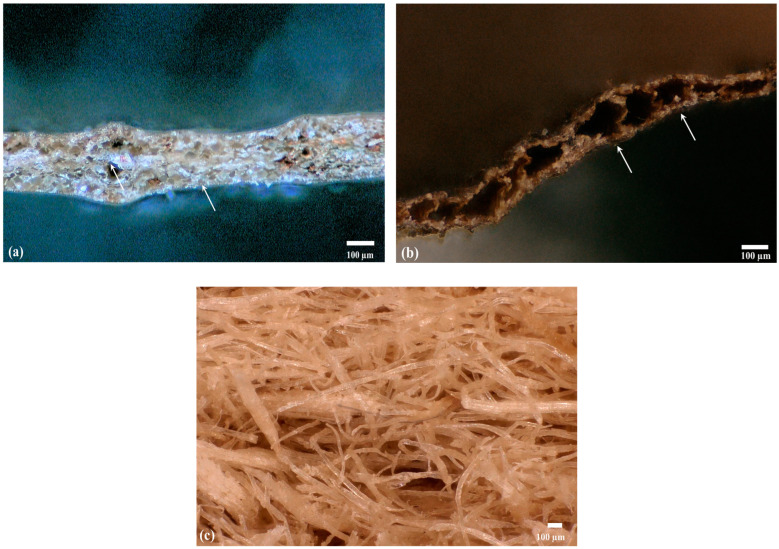
Microscopy images showing the internal structure of seagrass leaves. Cross-section view of *Posidonia oceanica* seagrass (**a**), *Zostera marina* seagrass (**b**), and wood fibers (**c**). The white arrows indicate the porous structure of leaves (closed cells).

Another cause for the low TC of POL and ZM boards is the internal porous and spongy structure of seagrass leaves. *Posidonia oceanica* seagrass leaves contain a high number of pores of various sizes (Figure 3a). The internal structure can also act as an insulation layer. *Zostera marina* leaves have a very similar structure to the former; the size of their pores, however, is much larger (Figure 3b). In the case of wood fibers (Figure 3c), heat flow takes place by conduction through the single fibers and through convection (air). The high porosity in the wood fiberboard structure results in a relatively low TC.

### 3.4. Fire Resistance

**Figure 4:**
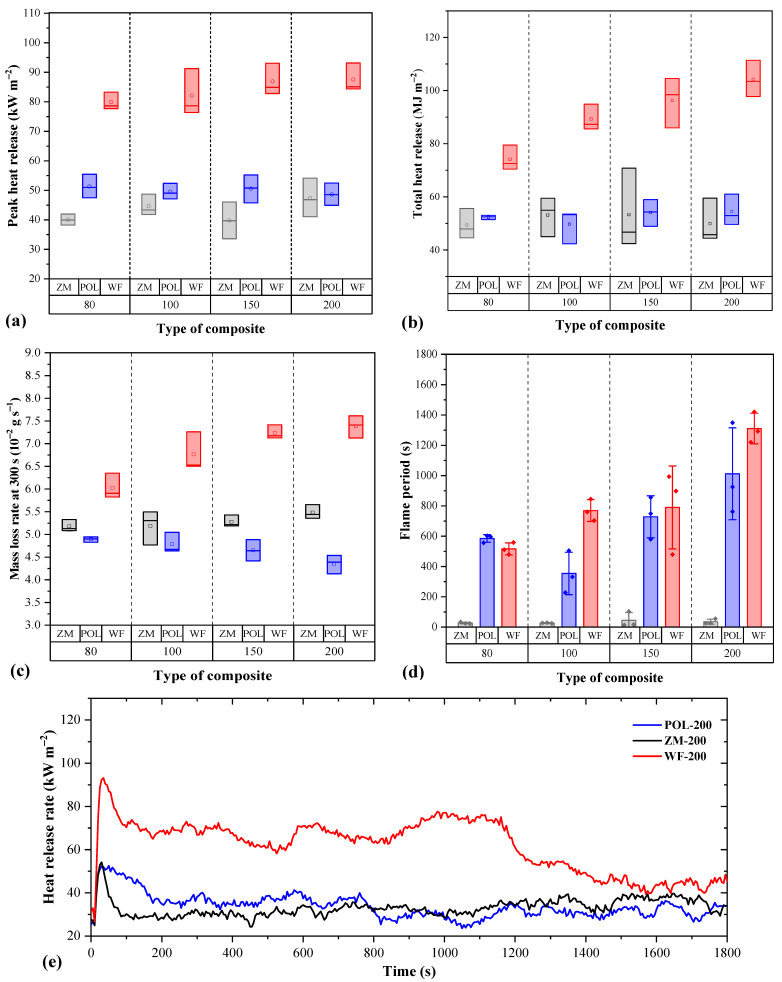
Fire properties of insulation boards of various densities determined with cone calorimetry. Peak heat release (**a**), total heat release for 1800 s (**b**), mass loss rate at the initial periods (300 s) (**c**), flame period (difference between ignition and flameout) (**d**), heat release rate for specimens with the target density of 200 kg m^−3^ (**e**). In plots (**a**–**c**), each box represents the standard deviation, the horizontal lines within the box represent the median, and the dots represent the mean value. In plot (**d**), the height of the column represents the mean value, the vertical line on each column represents the standard deviation, and the dots represent individual data.

Cone calorimetry, a method used in the field of fire safety engineering, showed that seagrass-based boards are much more resistant to fire compared to WF boards (Figure 4). Organic-based insulation materials are known to be susceptible to fire. All boards ignited within the first 10 s of being exposed to the heat. However, significant differences were noticed in terms of heat released during combustion, duration, and characteristics of the flame generated. The peak heat release (PHR) of POL and ZM boards was considerably lower compared to WF boards (Figure 4a). Specifically, WF boards had up to 70% higher PHR compared to POL and more than 110% higher compared to ZM boards. There was no visible change in PHR with increasing density. Similarly, the total heat release (THR) was twice as high for WF boards compared to seagrass boards (Figure 4b).

In contrast with PHR, the THR of WF boards increased with density. At high board densities, a large amount of the combustible organic mass is burned, resulting in high THR. Interestingly, no variance of THR with density was observed for seagrass-based boards. These were much more difficult to burn and exhibited a very weak flame after ignition. The mass loss rates at 300 s (MLR) were consistent with the THR and PHR (Figure 4c). A slight difference was observed between boards of POL and ZM. Although in the case of ZM boards the flame extinguished very quickly (after approximately 30 s), the MLR was higher compared to POL boards. For POL boards, MLR tended to decrease with density even though they develop a flame for a relatively long period (Figure 4d). The POL boards sustained the flame for long periods, but this did not seem to affect their mass. With increasing density, the lignocellulosic mass tended to burn for long periods. As density increases, the absence of large voids affects the MLR by decreasing it. In the case of the ZM boards, the lignocellulosic mass burned for only a few seconds; yet still a mass loss was observed even though there was no flame.

MRL of WF boards was significantly higher compared to seagrass-based boards, and increased with density. Tiny wood fibers are very susceptible to fire and burn easily followed by a great mass loss rate. In the case of the WF boards, a major portion of the heat was released in the first 300 s (Figure 4e).

The single flame test was carried out for two different periods, for 15 and 30 s at the surface and the edge, respectively. None of the seagrass-based boards ignited when the flame was applied (Table 5). On the other hand, all of the WF boards were ignited. The burned area (soot cone height) of seagrass-based boards varied from 27 to 35 mm when the flame was applied to the surface for 15 s. For the longer period of flame application, the cone height slightly increased. All WF board specimens ignited regardless of density. When the flame was applied on the edge of the specimen for 30 s, the burnt area was very large and exceeded the limit of 150 mm within a short period (less than 30 s after the burner is removed), resulting in failure of the test.

The high performance as related to flame and heat resistance of seagrass boards might be associated either with the structure or their chemical composition. The chemical analysis (Table 3) showed that seagrass leaves contain high amounts of mineral constituents (ash). Ash itself can be composed of silica (SiO_2_), sodium chloride (NaCl), and other trace minerals. As the flame of intense heat is applied to the specimens, a protective layer forms on their surface, which acts as an insulation layer for the inner organic material. Similar behavior has been observed in rice husk-based materials. The silica layer present in these materials can reradiate heat from an external heat source while insulating the unburnt mass, thus providing a sufficient shielding effect [40]. Owing to the flame-shielding effect, a possible application of seagrass leaves could be a fire protection coating. Owing to the broad leaf structure and the capability to insulate and protect against fire, layers of seagrass leaves can be attached to existing wooden structures to protect them from thermal flux. Additionally, their pleasant brownish appearance could be attractive for interior decoration.

Based on the results of the single flame test, we can estimate that the fire resistance class of seagrass-based insulation boards is B, C, or D. On the other hand, some of the WF boards did not pass the single flame tests, which means that they can be classified as class E. Hakkarainen [41] proposed that the fire class can be predicted based on the ignitability and the cone calorimetry results. Hakkarainen suggested that materials releasing less than 50 kW m^−2^ are predicted to be class A2/B. Most of the seagrass-based boards exhibited low-peak heat release (even lower than 50 kW m^−2^). However, Hakkarainen also stated that if the ignition time is shorter than 5 s or longer than 60 s, the specimens are outside the range of the index approach. Owing to the high uncertainty of the class prediction, this procedure was not applied in our case; in some of our samples, ignition occurred earlier than 5 s.

### 3.5. Water Absorption

**Figure 5:**
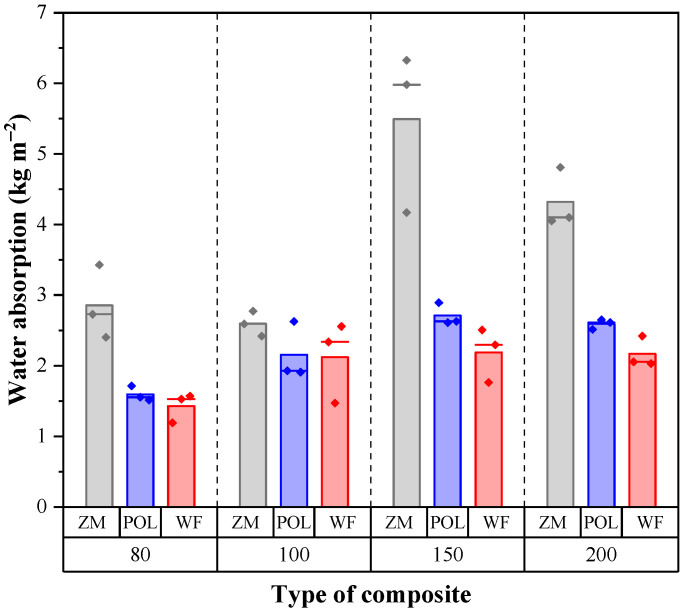
Water absorption of insulation boards of various densities. The height of the column represents the mean value, the vertical line on each column represents the standard deviation, and the dots represent individual data.

The water absorption of seagrass-based boards appears to be higher than that of WF boards (Figure 5). The latter differed slightly from POL and was considerably lower compared to ZM boards. The water absorption tended to increase with the density of the boards. The water-related properties of the boards depend on the morphological characteristic and chemistry of raw materials. Water can wet the surface of wood fibers in the WF board specimen. After the specimens are removed from the water container, the adhesive forces holding the water to the wood fibers are weaker than the gravity forces, so that the water flows downward and leaches out of the specimen. The pMDI binder can further hydrophobize the fiber surface, thereby reducing the adhesion forces of water to the fibers.

On the other hand, even though seagrass fibers have a smaller surface area, they contain pores (Figure 3). POL seagrass leaves contain many pores with a wide size distribution, while ZM leaves have few large pores. ZM boards absorbed far more water compared to POL boards. This difference might be associated with the capillary forces keeping water out of the pores. The far larger pores in ZM leaves have low capillary forces, so that much more water can be absorbed, resulting in high water absorption. Another factor that influences the water absorption is the chemical composition of the raw materials. Wood fibers contain higher amounts of lignin than seagrass (Table 3). Lignin is known to be a hydrophobic component, implying low water absorption.

### 3.6. A Simplified Cost Analysis and Comparison with Other Natural Resources

**Figure 6:**
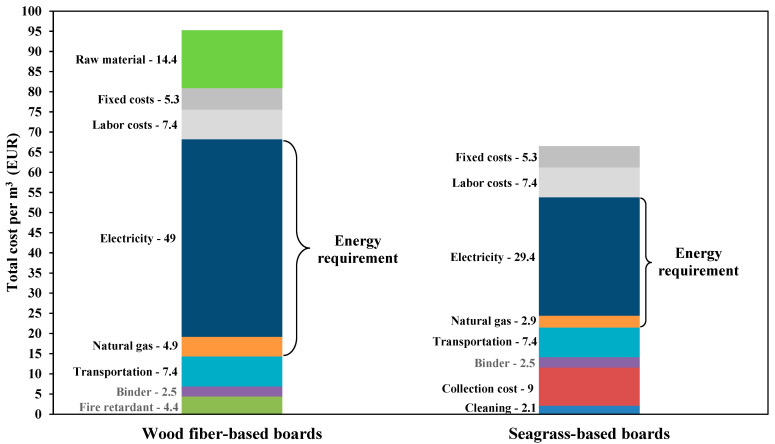
A simplified cost analysis of the insulation boards from seagrass leaves and wood fibers.

A simplified cost analysis was undertaken to estimate and compare the costs for the production of insulation boards from seagrass leaves and wood fibers. The economic analysis was carried out considering a target density of the insulation board of 200 kg m^−3^. For this analysis, we referred to the data of a previous study, conducted by Rocchi et al. [42]. The authors studied the use of pruning remains from *Tilia* wood to produce insulation boards. The cost data were adapted to our study. The individual costs were calculated for the year 2021, taking into account inflation rates (inflation rates of Germany). Prices for electricity prices (EUR kWh^−1^) and natural gas (EUR m^−3^) were estimated for July 2021, and the data were obtained from the European Commission and Ycharts, respectively [43,44]. As seagrass is a waste material, we did not include any initial cost for the raw material. The total cost of seagrass leaves consists of collection costs, transportation, cleaning operation, and processing of insulation boards. The cost of seagrass collection was obtained by Mainardis et al. [12]. The transport costs of the raw materials are assumed to be identical as they depend on the location of the processing plant. The binder cost, the fixed costs, and labor costs are also assumed to be similar as the same amount of binder can be used for spraying. The operating costs are also similar for the two types of raw materials. In terms of energy requirement, seagrass leaves do not need to be pre-heated and refined, which accounts for 40% of the total energy required for the production of insulation/MDF fiberboards [45,46]. These costs are excluded from seagrass processing as leaves might be used to produce insulation boards in their original form. Energy is only required for the initial drying and hot-pressing of the boards along with other side operations. Seagrass leaves might also need to be cleaned from sand particles by using a horizontal-vibrating sieving machine. To optimize this operation, the conveyor can be adapted (conveyor belt with holes), making it even more cost-effective. The costs for cleaning were estimated at 2 EUR m^−3^. Considering the high fire resistance of seagrass-based boards, the cost of adding fire retardants is eliminated. After calculating of the abovementioned single costs, the total cost of seagrass-based boards is estimated at 66.4 EUR m^−3^, while the cost of WF-based boards reaches 95.1 EUR m^−3^ (Figure 6). The obtained costs are within the range of estimated costs (37–145 EUR m^−3^, excluding the logistics) for wood-based insulation materials, mentioned in previous work [42].

Apart from the economical profitability, a comparison of seagrass with other natural fibers (and synthetic materials) shows the advantages of seagrass materials (Table 6). Comparing our results with those of other natural fibers, it seems that the TC is better (lower) or similar within the same density range. Some of the natural fibers reportedly have lower TC compared to seagrass. However, the densities of other fibers are very low, which might be an indication of the low mechanical properties of the respective composites. In terms of fire resistance, most of the other natural fibers are classified as class E. Rice husk and flax seem to be the most fire-resistant materials. Both flax and rice husk, however, have a relatively high TC (up to two times higher than seagrass) at high densities. The embodied (gray) energy is associated with the sum of the impacts of all greenhouse gas emissions attributed to the material during its life cycle. Some of the natural fibers mentioned are highly insulating materials, but generally require a high amount of energy to be harvested, processed, transported, etc. Seagrass leaves do not need to be heavily processed and therefore can potentially have low embodied energy. However, additional studies need to be conducted to verify this hypothesis.

## 4. Conclusions

The utilization of seagrass leaves from wracks in the manufacturing of insulation boards not only reduces greenhouse gas emissions but is also an effective measure to avoid costly landfilling. From the technical point of view, the boards comprising seagrass appear to be better thermal insulators than the corresponding WF boards. More specifically, the TC of WF boards is 5 to 12% higher than that of seagrass-based boards. The heat release during the combustion of the latter is twice as low compared to the former, indicating a very high fire resistance. In terms of mechanical properties, seagrass-based boards have lower compression and internal bond strength compared to boards made of wood fibers at the same range of densities. However, with seagrass boards, the strength requirements of building insulation materials can be met at densities varying between 150 and 200 kg m^−3^. The simplified cost analysis shows that seagrass leaves are an inexpensive and ecological alternative to wood fibers due to the low raw material costs, the low energy required for their processing, and the fact that they might require none to very low amounts of fire retardants. The use of seagrass leaves as an insulation material is one of the most effective practices in the management of seagrass wracks and does not compete with other management scenarios. Instead, this strategy can be an intermediate link in the waste management chain. After the end of their life as an insulation material, seagrass leaves can still be considered a valuable resource and could be further exploited for biogas production, composting, or energy generation.

## Figures and Tables

**Table 1:** Production variants and the respective abbreviations.

Raw Material	Amount of Raw Material (g)	Amount of Binder (g)	Target Density (kg m^−3^)	Type of Composite
*Zostera marina* leaves	818	43	80	ZM-80
	1023	54	100	ZM-100
	1534	82	150	ZM-150
	2045	109	200	ZM-200
*Posidonia oceanica* leaves	837	43	80	POL-80
	1046	54	100	POL-100
	1569	82	150	POL-150
	2092	109	200	POL-200
Wood fibers	789	43	80	WF-80
	989	54	100	WF-100
	1479	82	150	WF-150
	1973	109	200	WF-200

**Table 2:** Geodesic length and thickness distribution of seagrass Zostera marina (ZM), seagrass Posidonia oceanica (POL), and wood fibers (WF).

Percentile (%)	POL (Leaves)		ZM (Leaves)		WF (Fibers)	
	Geodesic Length (mm)	Width (mm)	Geodesic Length (mm)	Width (mm)	Geodesic Length (mm)	Thickness (mm)
0	5.23	1.03	0.10	0.04	0.05	0.02
10	48.39	3.68	13.48	0.78	0.63	0.05
50	78.97	5.71	36.74	1.56	2.12	0.06
90	94.32	7.04	72.22	3.10	4.61	0.27
100	99.68	8.09	86.01	4.29	16.61	1.65

**Table 3:** Chemical composition of raw lignocellulosic materials.

Raw Material	HWE ^a^ (%)	CEE ^b^ (%)	Lignin (%)	Holocellulose (%)	Ash (%)
ZM (leaves)	17.9	1.0	17.5	44.6	22.0
POL (leaves)	10.9	4.2	18.7	54.7	13.9
WF (fibers)	9.0	2.1	28.3	66.0	0.5

^a^ HWE indicates hot-water extractives, ^b^ CEE indicates cyclohexane–ethanol extractives.

**Table 4:** Internal bond strength and compression strength of produced composite boards.

Type of Composite	Actual Density (kg m^−3^)	Internal Bond Strength (kPa)	Compression at 10% Deformation, σ_10_ (kPa)
ZM-80	86 ± 5	2.8 ± 1.2	17.5 ± 6.3
ZM-100	103 ± 4	5.1 ± 1.0	24.1 ± 9.4
ZM-150	151 ± 4	8.0 ± 0.9	42.6 ± 12.1
ZM-200	206 ± 9	9.4 ± 3	102.7 ± 48.4
POL-80	83 ± 8	2.6 ± 1.3	13.9 ± 3.6
POL-100	103 ± 6	4.5 ± 1.4	15.9 ± 1.2
POL-150	152 ± 7	6.7 ± 4.3	38.4 ± 3.1
POL-200	209 ± 13	21.0 ± 6.0	95.4 ± 15.0
WF-80	81 ± 3	10.6 ± 1.4	35.4 ± 2.6
WF-100	104 ± 2	16.4 ± 1.5	56.2 ± 2.1
WF-150	154 ± 5	29.5 ± 4.7	151.0 ± 5.8
WF-200	202 ± 6	42.8 ± 6.1	254.4 ± 17.3

Mean values and (±) standard deviations.

**Table 5:** Single flame test results for produced seagrass-based and WF insulation boards.

Type of Composite	Bunsen Burner Analysis at the Surface for 15 s				Bunsen Burner Analysis at the Edge for 30 s			
	Ignition	Time to Flameout (s)	Soot Cone Height (mm)	Pass/Fail *	Ignition	Time to Flameout (s)	Soot Cone Height (mm)	Pass/Fail **
ZM-80	No	n/a	35	Pass	No	n/a	32	Pass
ZM-100	No	n/a	27	Pass	No	n/a	30	Pass
ZM-150	No	n/a	27	Pass	No	n/a	41	Pass
ZM-200	No	n/a	28	Pass	No	n/a	41	Pass
POL-80	No	n/a	35	Pass	No	n/a	47	Pass
POL-100	No	n/a	32	Pass	No	n/a	55	Pass
POL-150	No	n/a	31	Pass	No	n/a	51	Pass
POL-200	No	n/a	31	Pass	No	n/a	38	Pass
WF-80	Yes	19	118	Pass	Yes	>60	All ***	Fail
WF-100	Yes	60	110	Pass	Yes	>60	All ***	Fail
WF-150	Yes	28	90	Pass	Yes	44	112	Pass
WF-200	Yes	49	All ***	Pass	Yes	>60	All ***	Fail

Each measurement was performed in duplicate. * The test is regarded as pass when the flame extinguishes within 15 s after burner is removed without passing the cone height of 150 mm. ** The test is regarded as pass when the flame extinguishes within 30 s after burner is removed without passing the cone height of 150 mm. *** The entire surface of the specimen is burned.

**Table 6:** The thermal conductivity, resistance to fire, and the embodied energy for various types of insulation at a specific range of densities.

Insulation Type	Density (kg m^−3^)	Thermal Conductivity (W m^−1^ K^−1^)	Resistance to Fire	Embodied Energy (MJ kg^−1^)	References
EPS	18–50	0.029–0.041	E	80.8–127	[47]
Flax	20–100	0.033–0.090	C	39.5	[47,48]
Hemp	25–100	0.039–0.123	E	18.7	[48]
Kenaf	30–180	0.026–0.044	E	22.7–39.1	[48]
Rice husk	130–170	0.048–0.080	C	1.4	[48,49]
Date palm	187–389	0.072–0.085	n/a	n/a	[50]
Coir fibers	50–160	0.040–0.050	D-E	0.55	[48]
Cotton stalks	150–450	0.058–0.082	E	44–48	[47,51]
Wood fibers	50–270	0.040–0.052	E	20.3	[47,52]
Seagrass POF *	70–130	0.037–0.043	n/a	n/a	[52]
Wood fibers	92–212	0.044–0.057	E	n/a	Current study
Seagrass POL *	95–233	0.042–0.050	B-D	n/a	Current study
Seagrass ZM *	83–213	0.042–0.051	B-D	n/a	Current study

* POF indicates *Posidonia oceanica* fibers (balls), POL indicates *Posidonia oceanica* leaves, and ZM indicates *Zostera marina* leaves.

## Data Availability

The data presented in this study are available upon request from the corresponding author.
